# Controlling hemoptysis: An alternative approach

**DOI:** 10.4103/0970-2113.71981

**Published:** 2010

**Authors:** M. S. Barthwal

**Affiliations:** *Department of Pulmonary, Critical & Sleep Medicine, Military Hospital (Cardiothoracic Centre), Pune, Maharashtra, India. E-mail: msbarthwal@rediffmail.com*

Sir,

I read with interest the case report titled “Controlling hemoptysis: An alternative approach” published in Lung India 2010;27:99-101.[1] I would like to offer following comments:

- The title of the case report suggests that there is some novel alternative approach in controlling hemoptysis. On the contrary, authors have discussed bronchial arterial embolization (BAE) which is in neither an alternative nor a novel approach. Authors have in fact tried to highlight the role of prophylactic BAE before bronchial biopsy of lesions which are likely to result in post procedure uncontrolled/ massive hemoptysis.
- The reported case was finally diagnosed as squamous cell bronchogenic carcinoma on bronchial biopsy. Authors decided to do BAE before bronchial biopsy since the same was advised by radiologist based on the findings of vascular nature of tumor. The following observations about the approach followed in work up of this case need justification from the authors:
  - The CT findings described do not suggest vascular nature of mass and even if radiologist had doubts about its vascular nature, they should have advised CT angio to further delineate and confirm the nature of vascular tumor. It is not understood that how come BAE was decided in this case before bronchial biopsy.
  - There are no indications of doing prophylactic BAE before biopsy of any endobronchial lesions and the same has not yet been reported in literature. Authors also have not discussed the same.
- The discussion mainly comprises indications, procedure details and complications of BAE. Authors should have instead discussed the prophylactic role of BAE, if any, before bronchoscopy.
